# Protective Effect of a (Poly)phenol-Rich Extract Derived from Sweet Cherries Culls against Oxidative Cell Damage

**DOI:** 10.3390/molecules21040406

**Published:** 2016-03-24

**Authors:** Ana A. Matias, Rita Rosado-Ramos, Sara L. Nunes, Inês Figueira, Ana Teresa Serra, Maria R. Bronze, Claúdia N. Santos, Catarina M. M. Duarte

**Affiliations:** 1 Instituto de Tecnologia Química e Biológica António Xavier, Universidade Nova de Lisboa, Avenida da República, Estação Agronómica Nacional, Oeiras 2780-157, Portugal; ritajoao.ramos@gmail.com (R.R.-R.); sara.nunes88@gmail.com (S.L.N.); inesf@itqb.unl.pt (I.F.); tserra@itqb.unl.pt (A.T.S.); mrbronze@itqb.unl.pt (M.R.B.); csantos@itqb.unl.pt (C.N.S.); cduarte@ibet.pt (C.M.M.D.); 2 Instituto de Biologia Experimental e Tecnológica, Avenida da República, Quinta-do-Marquês, Estação Agronómica Nacional, Apartado 12, Oeiras 2781-901, Portugal; 3 Instituto de Investigação do Medicamento (IMED), Faculdade de Farmácia da Universidade de Lisboa, Avenida das Forças Armadas, 1649-019 Lisboa, Portugal

**Keywords:** polyphenols, Saco cherry, oxidative stress, antioxidant, intestinal epithelium, neurodegeneration

## Abstract

Oxidative stress is one of the key phenomena behind the most common types of chronic diseases. Therefore, the modulation of oxidative stress is an interesting target for acting either through prevention or as a therapeutic approach. In this work, a Portuguese variety of cherry (Saco Cherry) was processed in order to obtain a potent *in vitro* antioxidant phenolic-rich extract (Ch-PRE), which was further explored to evaluate its potential application as nutraceutical agent against cellular oxidative stress damage. Ch-PRE was mainly composed of anthocyanins, particularly cyanidin-3-rutinoside, cyanidin-3-glucoside, peonidin-3-glucoside and neochlorogenic acid, and exhibited a potent chemical antioxidant activity expressed by its oxygen radical absorbance capacity (ORAC) and hydroxyl radical averting capacity (HORAC) values. Ch-PRE also displayed effective intracellular radical scavenging properties in intestinal epithelial and neuronal cells challenged with oxidative stress but showed a different order of effectiveness regarding the modulation of endogenous antioxidant system. Ch-PRE could be an attractive candidate to formulate an agent for the prevention of oxidative stress-induced disorders such as intestinal inflammation disorders or with an appropriated delivery system for neurodegenerative diseases.

## 1. Introduction

Oxidative stress is characterized by an unbalance in reactive oxygen species (ROS) production and the antioxidant defense system that leads to an intracellular accumulation of these harmful radicals [1,2,3]. Consequently, important cellular biomolecules such as proteins, lipids and DNA are damaged by ROS leading to a deficient or complete loss of function of these molecules with deleterious consequences [2]. In this context, oxidative stress is pointed as an important and common mechanism underlying most known types of chronic and degenerative diseases such as diabetes, cardiovascular diseases, chronic intestinal inflammation, cancer and neurodegenerative disorders. Thus, the modulation of oxidative stress rises as a relevant target for prevention or treatment of these diseases [3,4,5,6].

Concerning chronic intestinal inflammation, ROS can interfere with signal transduction in many pathways leading to alterations in gene expression and they are also able to disrupt intestinal barrier integrity contributing to the inflammatory condition and also for the overstated immune response present in this disease [4,5,6]. Furthermore, recent studies show that patients with chronic intestinal inflammation have a low activity of endogenous antioxidant systems, which reinforce the importance of oxidative stress in these diseases [5,7].

Concerning neurodegenerative diseases, like Alzheimer’s disease (AD) and Parkinson’s disease (PD), there are strong evidences demonstrating that several ROS-mediated pathways may be involved in the degeneration process. It has been described that the accumulation of iron in the brain leads to higher ROS generation, involvement of mitochondrial pathways and to a decrease of endogenous antioxidants levels [8].

Cellular endogenous antioxidants, include enzymes that are responsible for the reduction of ROS and also encompass the glutathione system. Exogenous antioxidants are obtained from diet and include phytochemicals such as (poly)phenols. (Poly)phenols can act not only as direct antioxidants by preventing or scavenging ROS but also as metal chelators and modulators of cellular pathways including the modulation of the endogenous antioxidant systems [1,2,3,9].

Anthocyanins are a class of (poly)phenols responsible for the red color of several fruits and are considered as the most potent natural antioxidants [10,11]. Due to their characteristic molecular structure, anthocyanins are able to directly scavenge ROS or prevent their formation through metal chelation properties [12].

*Prunus avium* L. (sweet cherry) are widely consumed fruits which constitute a rich source of (poly)phenolic compounds, in particular anthocyanins [10,13,14]. In Portugal, the annual production of cherries is about 17,000 tons. Among all cherries, some cultivars are not well appreciated by the consumer due to their small size being in part considered as a food supply chain waste that are currently not properly taken care of, both in environmental and economic terms [15,16,17,18]. One example is Saco cultivar, an old traditional cherry, only cultivated in specific regions of Portugal. This agro-biomaterial (biomass) is an ultimate substrate for the recapture of functional and bioactive compounds, (in particular (poly)phenols, terpenes and soluble fibers) and for the development of new products with high market value [14,18,19].

Recovery of (poly)phenols from plant crude extracts by adsorption is a process in expansion in the nutraceutical market [20,21]. The application of synthetic adsorbent materials like macroporous resins has several advantages, such as low operation costs, simple handling, and high adsorption capacities [20]. The use of commercial adsorbents for food production purposes is regulated by the US Food and Drug Administration and European Commission. Isolation of (poly)phenols and anthocyanins from natural sources using adsorption lead to extracts with higher concentrations in these compounds as well antioxidant, chemotherapeutical and anti-inflammatory activities [22,23,24,25].

In our previous work, we reported that Saco cherry cultivar possess high phenolic content, namely in anthocyanins, and antioxidant capacity when compared with other cherry cultivars [13], emphasizing their potential for further industrial exploitation and valorization as a rich source of high added-value compounds. In this field, high-pressure methodologies have already been investigated to obtain cherry extracts rich in bioactive compounds with antioxidant and antiproliferative effect [18,19].

Taking all this into account, the aim of this work was to explore the application of a green separation process to develop a phenolic-rich extract from Saco cherry culls (Ch-PRE) and assess its bioactivity against different oxidative-stress cell models. Ch-PRE was obtained through a batch adsorption process, using a food grade macroporous resin and analyzed for their phenolic composition and antioxidant activity using cell-free assays. The intracellular antioxidant activity was also evaluated on intestinal epithelial cells (Caco-2 cells) and neuronal cells (SK-N-MC cells), selected as preclinical models for chronic intestinal inflammation and neurodegenerative diseases, respectively.

## 2. Results and Discussion

A phenolic-rich fraction (Ch-PRE) was prepared, from non-marketed Saco cultivar cherries aiming at developing added-value ingredients against oxidative stress. A two-step separation process was applied: first step encompasses a solid–liquid extraction with a 1:1 mixture of ethanol:water followed by an adsorption process performed in batch mode with a macroporous resin Amberlite^®^ XAD-16. The adsorption step allows the isolation of the phenolic fraction from the remaining constituents, like terpenes and fibers. The (poly)phenolic fraction of hydro-alcoholic extract was adsorbed on the polymeric resin and the majority of other constituents such as carbohydrates, organic acids and minerals were discharged on supernatant. The resulting extract, the desorbed fraction (Ch-PRE), were further evaluated in terms of (poly)phenolic composition and antioxidant capacity using non-cellular and cell-based assays.

### 2.1. Characterization of Ch-PRE

**Figure 1:**
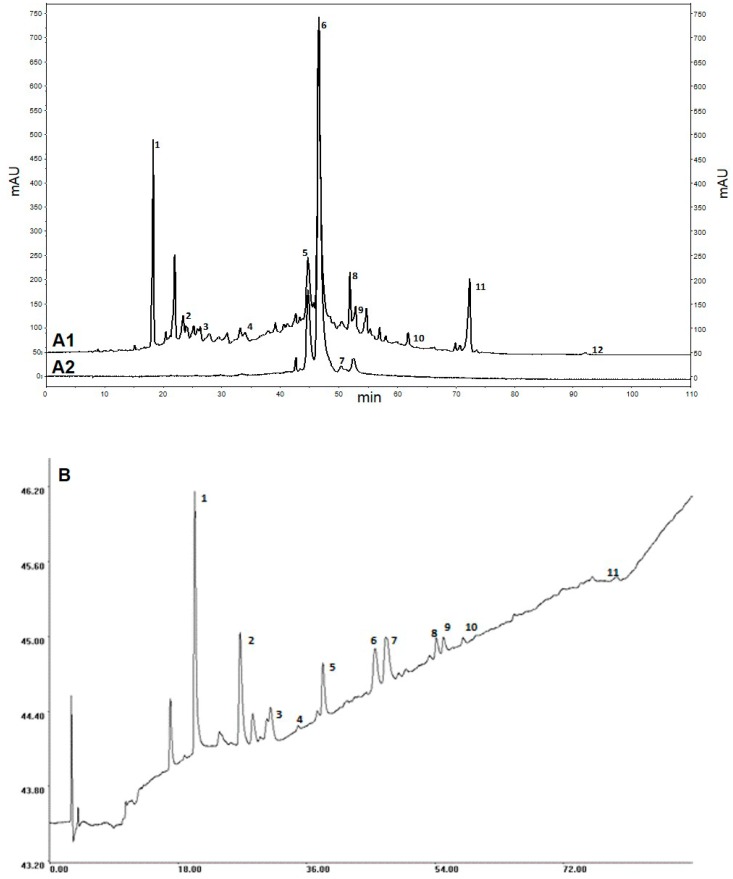
Chromatographic profiles of Ch-PRE (**A1**) High Performance Liquid Chromatography coupled with a diode-array detector (HPLC-DAD) profile at 280 nm; (**A2**) HPLC-DAD profile at 527 nm; and (**B**) HPLC coupled with a DAD and electrochemical detector (ED) (HPLC-DAD-ED). Legend: 1—Neochlorogenic acid, 2—Catechin, 3—Chlorogenic acid, 4—Procyanidin B2, 5—Cyanidin-3-glucoside, 6—Cyanidin-3-rutinoside, 7—Peonidin-3-glucoside, 8—Quercetin-3-rutinoside, 9—Quercetin-3-glucoside, 10—Kaempferol-3-glucoside, 11—Sakuranin, 12—Isosakuranetin.

Ch-PRE was characterized for phenolic composition using chromatographic techniques in tandem with diode array and electrochemical detectors (Figure 1). Results showed that, in the end of the process, Ch-PRE contained approximately 50% (*w*/*w*) of (poly)phenols (456.9 ± 13.8 mg of gallic acid equivalents per g of dry extract—mg GAE·g^−1^ dw) representing a ~45-fold increase compared to (poly)phenolic content of the cherries hydro-alcoholic extract (data not shown). The main compounds present in Ch-PRE are anthocyanins (153.94 mg cyanidin-3-rutinoside equivalents g^−1^ dw), particularly cyanidin-3-rutinoside, cyanidin-3-glucoside, and peonidin-3-glucoside and neochlorogenic acid, a phenolic acid (Figure 1 A, Table 1 and Table 2). The presence of other phenolic acids and flavonoids such as chlorogenic acid, catechin, procyanidin B2, rutin, quercetin-3-glucoside and kaempferol-3-glucoside, was also detected (Figure 1A and Table 1). These compounds have already been identified in several sweet cherries cultivars by Serra and co-workers [13] and their concentration are up to 30-fold lower than in Ch-PRE.

Sakuranin and its glycoside derivate isosakuranetin were also identified in Ch-PRE by MS/MS (Table 1). These two compounds have already been identified in Saco cherry’s extracts obtained by supercritical carbon dioxide and pressurized liquid extraction [18]. The three main anthocyanins were identified and quantified by HPLC-DAD-ED using standards (Table 2).

Antioxidant activity of Ch-PRE was first evaluated using two different but complementary *in vitro* chemical assays: ORAC assay measures peroxyl radical scavenging capacity and HORAC assay primarily reflects a capacity against hydrophilic chain-breaking hydroxyl radicals. Ch-PRE possesses a high ability to scavenge peroxyl radicals (expressed by ORAC value: 7611 ± 213 μmol TEAC g^−1^·dw) as well as in inhibiting hydroxyl radical formation (expressed by HORAC value: 6874 ± 584 μmol CAEAC g^−1^·dw). It is important to note that ORAC of Ch-PRE is about two-fold higher than Vitamin C (3220 ± 312 μmol TEAC g^−1^·dw) [26] a well know antioxidant compound. The high HORAC value determined for Ch-PRE, comparing with other (poly)phenols-rich extracts, in particular cactus pear and apple extracts [24,25], may be a result of its high content in both anthocyanins and neochlorogenic acid. Anthocyanins are known to protect against the formation of both types of radicals (peroxyl and hydroxyl). The scavenge capacity is due to their ability to donate a hydrogen atom from an aromatic hydroxyl group to a free radical and their singular molecular structure allows the chelation of metal ions involved in Fenton or Fenton-like reactions reducing the hydroxyl radicals formation [12,14]. Moreover, the phenolic compounds identified in our study have already been described as potent active antioxidant species present in cherries; Piccolella and collaborators [27] shows that flavonoids like catechin, anthocyanins as well as hydroxycinnamic acids, such as chlorogenic and neochlorogenic acid, are the most active compounds in scavenging free radicals in sour cherries. Electrochemical detection (ED) is recognized as an important tool to study and identify compounds with antioxidant activity [13,28]. Peaks detected by the electrochemical detector correspond to reactive species with strong capacity to donate electrons [28,29]. Therefore, a positive relation between ED peaks and the antioxidant properties of the extract is likely to be found. Herein, the ED spectrum of Ch-PRE (Figure 1B) was performed and all identified compounds (Figure 1), with the exception of isosakuranetin which is present in low concentration, were also detected in the electrochemical spectrum, highlighting the antioxidant profile of this extract.

### 2.2. Protective Effect of Ch-PRE in Human Intestinal Epithelial Cells

Caco-2 cells are a suitable model of intestinal epithelium, since after differentiation, it forms a monolayer that mimics several characteristics of intestinal epithelial cells (IECs). It reproduces the main features such as defined tight junctions between cells, formation of microvillus at the apical cell surface and expression of many brush-border proteins including digestive enzymes, transporters and receptors [30]. In our study, the protective effect of Ch-PRE against oxidative stress at intestinal level was then evaluated using this human cell-based model of IECs. The cytotoxicity of Ch-PRE extracts in IECs was evaluated by testing a range of concentrations during 48 h. Cellular viability was not affected by the exposure to Ch-PRE (data not shown) and the concentration of 50 µg GAE·mL^−1^ was selected to be used in all subsequent assays. This Ch-PRE concentration was chosen since this is a predictable physiological concentration of (poly)phenols reaching intestine after digestion [31].

**Figure 2:**
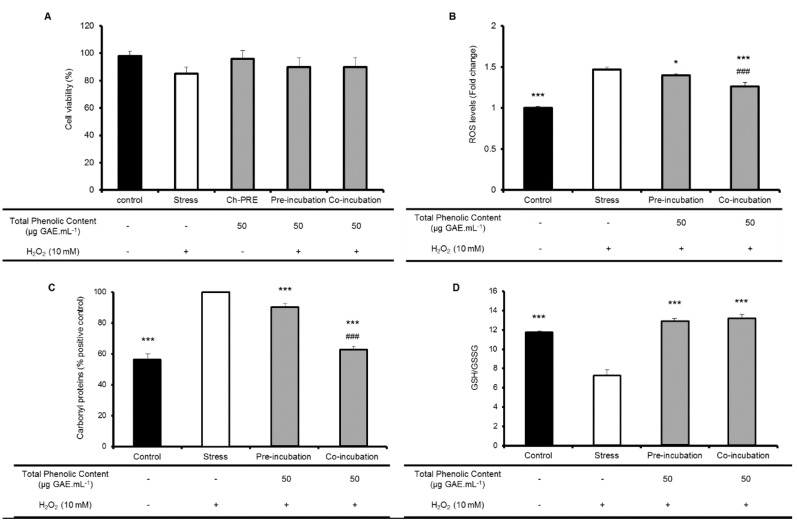
Cell viability upon H_2_O_2_ stress induction (10 mM) and for pre- and co-incubation of Ch-PRE (50 µg GAE·mL^−1^) in Caco-2 cells (**A**). Intracellular antioxidant activity in Caco-2 cells. Comparison between co- and pre-incubation treatments and effects of incubation with Ch-PRE (50 µg GAE·mL^−1^) during 1 h on: (**B**) ROS levels measured by DCFH-DA oxidation upon H_2_O_2_ stress induction (10 mM); (**C**) protein oxidation (stress inducer H_2_O_2_); and (**D**) ratio between GSH and its oxidized form, GSSG (stress inducer H_2_O_2_). Control represents cells non-challenged with stress inducers or with Ch-PRE; Stress represents cells challenged with stress inducer and not treated with Ch-PRE. Statistical differences between stress and control or cells treated with Ch-PRE are denoted as * *p* < 0.05 and *** *p* < 0.001; statistical differences between pre- and co-incubation treatments are denoted as ### *p* < 0.001. All values are means of three independent experiments ± SD.

Biomarkers of oxidative stress were assessed for evaluation of Ch-PRE bioactivity: ROS production; glutathione homeostasis and proteins carbonyl content (Figure 2). The assessment of these biomarkers was performed upon oxidative stress induction by H_2_O_2_ and through the evaluation of two different relevant physiological conditions, pre- and co-incubation of Ch-PRE with stress inducer. In this stress condition (10 mM H_2_O_2_, 1 h), cell viability is not affected, although ROS levels are boosted. In fact, none of the conditions (stress; pre-incubation of Ch-PRE and stress; stress; and co-incubation with Ch-PRE) affect significantly cellular viability (Figure 2A). The pre-incubation intends to mimic the preventive action of Ch-PRE when administrated regularly as an oral supplement. The co-incubation intends to simulate a therapeutic approach (with manifest disease).

The effectiveness of Ch-PRE in the reduction of intracellular ROS production was assessed after treatment with H_2_O_2_. The formation of intracellular ROS was monitored using the probe, DCFH-DA. Ch-PRE demonstrated to be able to significantly inhibit the formation or directly scavenge ROS-induced radicals (H_2_O_2-_induced) in both treatments applied (Figure 2B).

Indeed, Ch-PRE was more effective in the co-incubation approach (*p* < 0.001 relative to stress cells). This observation may be related with Ch-PRE anthocyanins content. Despite anthocyanins are able to counteract hydroxyl radicals, those compounds are poorly internalized by Caco-2 monolayer [32]. If anthocyanins, the major (poly)phenolic fraction present in Ch-PRE, are not taken up by intestinal epithelial cells, their contribution for ROS inhibition in pre-incubation should not be significant. In this case, the cellular antioxidant protection would be carried out more exhaustively by phenolic compounds with low molecular weight, such as neochlorogenic acid, that would be internalized by cells [33,34,35,36]. In contrast, in co-incubation treatment, anthocyanins are contemporarily with oxidative stress inducers and would act as extracellular free radical-scavenger or metal chelator, explaining the observed differences between treatments (*p* < 0.001).

We have previously shown that phenolic compounds with low molecular weight, catechin, chlorogenic acid and quercetin-3-rutinoside, had a positive correlation with inhibition of hydroxyl radical formation in Caco-2 cells after pre- or co-incubation with organic solvent-extracts of several cherries cultivars [13]. Moreover, in the same study, neochlorogenic acid and anthocyanins had a positive correlation in oxidative stress inhibition in co-incubation treatment [13].

The same significant difference (*p* < 0.001) between pre- and co-incubation treatment with Ch-PRE was also observed for protein oxidation alterations (Figure 2C). Protein carbonyls are a biological marker of oxidative stress and can be measured by the formation of carbonyl proteins [37]. The co-incubation of Ch-PRE with the stress inducer, when compared with non-stressed cells, causes a total attenuation in the production of protein carbonyls (*p* < 0.001) while the pre-incubation only reduce to some extend the oxidized proteins (*p* < 0.001). Once again, the surrounding environment of the cells seems to play a role in the cellular protection against the injuries promoted by oxidative stress. The presence on the extracellular milieu of anthocyanins and other compounds, may contribute to intestinal cellular protection through the modulation of the oxidative environment or even by further improving cellular defenses against oxidative injury.

Concerning the activity of Ch-PRE on the modulation of glutathione metabolism (Figure 2D) both treatments studied were able to restore GSH/GSSG ratio to the basal levels. Glutathione in its reduced form is one of the most important body’s endogenous antioxidant and its modulation by (poly)phenols or other phytochemicals may be very important to protect cells against oxidative aggressions [38]. Our results suggest that modulation of GSH system by Ch-PRE compounds taken up during pre-incubation promotes an intracellular protective effect even after stress treatment. The restoring of the ratio GSH/GSSG by Ch-PRE could be achieved by the modulation of enzymes from glutathione system (e.g., increase synthesis of GSH or GSH recycling by glutathione reductase), a mechanism already described to be associated in the recovery of redox homeostasis [38,39].

As far as we know, this is the first time that a cherry’s extract obtained using green separation processes revealed to be efficient in preventing oxidative damage on intestinal epithelial cells by modulating endogenous antioxidant system and reducing/attenuating protein oxidation. Protein oxidation is currently a suitable and accepted biomarker useful to substantiate health claims regarding antioxidant properties. Indeed, previous reports have shown that anthocyanins were able to avoid protein oxidation in similar situations but using *in vitro* chemical assays [40].

Taken together, oxidative intestinal epithelial cell damage was reduced by Ch-PRE and it was observed that cherry’s (poly)phenols are not only able to scavenge/inhibit ROS, protecting cells from oxidative stress, but they are also able to modulate cellular endogenous antioxidant system and regulate cell redox status at a predictable physiological concentration.

### 2.3. Protective Effect of Ch-PRE against Oxidative Stress in a Neurodegeneration Cell-Model

Although extracts rich in (poly)phenols may exert a positive effect in oxidative stress, not every (poly)phenols are able to cross the Blood-Brain-Barrier and reach the neuronal cells. Anthocyanins have been detected in the hippocampus but the concentrations are almost untraceable. Nonetheless, a wide range of (poly)phenols are able to reach the brain in different degrees and depending on their structure [41,42]. Having in mind that the delivery issue can be afforded by a nutraceutical delivery formulation, the neuroprotective potential of Ch-PRE emerges as a relevant question.

SK-N-MC cells challenged with H_2_O_2_ for 24 h were used as neurodegeneration cell model. The exposure to low concentrations of H_2_O_2_ induces changes in cell metabolism leading to cell death, reproducing what may occur during neurodegenerative process [43]. The reactive oxygen species generated during a H_2_O_2_ exposure are related with ageing associated diseases [44,45]. Neuronal cells were treated with 300 µM H_2_O_2_ for 24 h, which reduced cell viability to around 50% as previously described in SK-N-MC cells by a flow cytometry analysis [46] and confirmed in this work in the viability test.

Previously, a cytotoxicity assay was conducted in order to determine the Ch-PRE not-toxic concentrations range to perform the neuroprotective assay. Concentrations ranging from 0 to 250 µg GAE·mL^−1^ were tested, and below 16 µg GAE·mL^−1^ there was no significant alteration in cell viability. Ch-PRE non-toxic concentrations (0.25, 0.5, and 1.0 μg GAE·mL^−1^) were selected since these levels are near the physiological concentrations 

As a pre-incubation treatment was performed, these results suggest that Ch-PRE contains phytochemical compounds that could be uptake by cells or fixed on cell membranes, with potential to be explored in a neuroprotection perspective. However, the mechanism(s) by which those metabolites exert a beneficial effect are unknown. Therefore, to better understand possible mechanisms behind the neuroprotection detected, some cellular present in human plasma (0–4 µM) [46]. Moreover it is described that (poly)phenols are able to reach the brain in lower concentrations [47].

**Figure 3:**
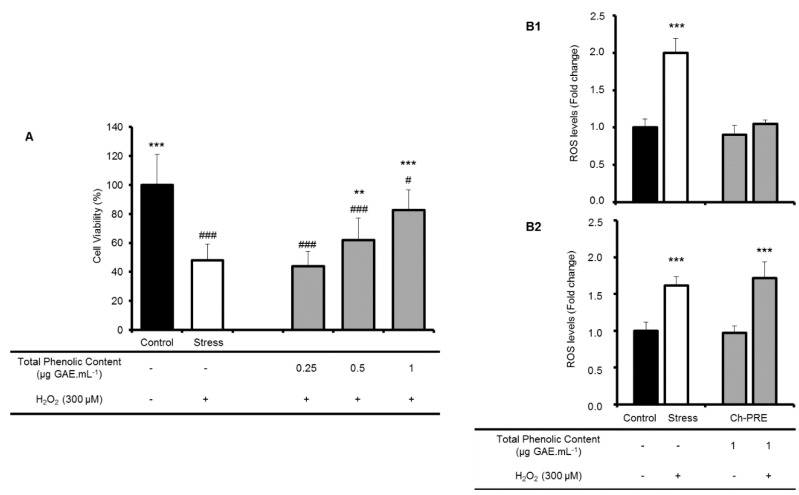
Neuroprotection exerted by Ch-PRE in SK-N-MC cells: (**A**) neuroprotective effect of Ch-PRE. Cell viability is expressed as percentage of viable cells. SK-N-MC cells were pre-incubated with 0.25, 0.5 and 1 µg GAE·mL^−1^ of Ch-PRE for 24 h and then injured with 300 µM H_2_O_2_ for 24 h. Control represents cells non-challenged with stress inducers or with Ch-PRE; Stress represents cells challenged with stress inducer and not treated with Ch-PRE. Statistical differences between control or treated cells and stressed cells are denoted as ** *p* < 0.01, *** *p* < 0.001 and statistical differences between treatments and control (not-treated cells) are denoted as # *p* < 0.05 and ### *p* < 0.001; (**B**) Relative ROS production by SK-N-MC cells pre-incubated with Ch-PRE for (**B1**) 2 h and (**B2**) 24 h and submitted to an oxidative stress (300 µM H_2_O_2_ for 30 min). Statistical differences in relation with cells not treated are denoted as *** *p* < 0.001. All values are means of three independent experiments ± SD.

Using the non-toxic concentrations of Ch-PRE, the neuroprotective capacity after incubation during 24 h, to mimic a daily dose administered, was evaluated by monitoring cell viability (Figure 3A). Interestingly a dose-dependent effect was observed, although there is not a complete rescue of cell viability after pre-incubation with Ch-PRE, a clear neuroprotection with Ch-PRE was observed at the highest tested concentration (1.0 μg GAE·mL^−1^). As a pre-incubation treatment was performed, these results suggest Ch-PRE contains phytochemical compounds that could be taken up by cells redox mechanisms were studied. The concentration that shows a higher neuroprotective effect (1 μg GAE·mL^−1^) was further assessed for intracellular antioxidant capacity in SK-N-MC cells.

The intracellular ROS levels were monitored (Figure 3B) in a complete different assay with cells challenged with H_2_O_2_ for only 30 min. In this situation cell viability was not affected although ROS levels increased. Two different times of Ch-PRE pre-incubation, 2 h (Figure 3B1) and 24 h (Figure 3B2), were performed to cover different time events. Within 2 h, direct scavenging effects from Ch-PRE can be determined, and at 24 h, indirect scavenging resulting from activation/modulation of other molecular mechanisms can be assessed. We assume that in a timescale of 2 h we can only detect an effect through scavenging properties of the polyphenols (direct effect) and when assessing the effect after 24 h of incubation other indirect mechanisms such as modulation of thiol-containing molecules (such as glutathione), ROS-inactivating enzymes or phase II detoxifying enzymes could be depicted [43].

In the absence of the oxidative stress, Ch-PRE incubation does not affect ROS levels (Figure 3B). When cells were challenged with a mild stress of 300 µM for 30 min, a significant increase on intracellular ROS was observed (100% relative to control without stress, Figure 3B) without promoting cell death. This effect was already described in a previous work, where cell membrane integrity was monitored by flow cytometry with propidium iodide [46]. Interestingly, a 2 h pre-incubation with Ch-PRE significantly reduced the intracellular ROS accumulation (*p* < 0.001), maintaining ROS balance at basal level. This effect is not detected at 24 h of Ch-PRE pre-treatment suggesting a possible preventive role of Ch-PRE against oxidative stress mediated by activities related with direct ROS production/scavenging (Figure 3B) and not particularly due to modulation of other antioxidant endogenous mechanisms.

These results are in agreement with previous work on various flavonoids (e.g., naringenin, nobiletin, luteolin, and quercetin) and their role on the molecular mechanism involved in neuroprotection as neuronal oxidative stress [48,49]. Moreover, anthocyanins, the main components of Ch-PRE, are reported as neuroprotective agents due to their direct antioxidant (scavenging) activity [50] but also by their ability to modulate transcription factors, gene expression, intracellular cell signaling, and also by their capacity to regulate the protein degradation [51]. More recently, cyanidin-3-*O*-glucoside, isolated and purified from tart cherries displayed neuroprotective properties against permanent middle cerebral artery occlusion [52]. The authors concluded that this protection is mediated in part by blocking the apoptosis-inducing factor release from mitochondria under oxidative stress [52].

In summary, the 2 h pre-treatment with Ch-PRE effectively alleviated oxidative stress in SK-N-MC cells caused by H_2_O_2_-induced injury, turning this extract attractive to be formulated and delivered to the brain in more complex interventions for neural disorders. However, further studies are necessary to deeply understand the mechanisms, doses and role of each Ch-PRE compound on neuroprotective activity (structure-activity relationship).

The beneficial effect of some isolated phytochemicals was described as achieved through the increment of antioxidant enzymes or protein chaperons as well as through the hormetic pathways [53]. Therefore, these molecular targets should be a goal for future studies into the neuroprotection mediated by Ch-PRE and its main constituents.

## 3. Experimental Section

### 3.1. Reagents

Methanol, 2′,2′-azobis (2-amidinopropane) dihydrochloride (AAPH), 2′,7′-dichlorofluorescein diacetate (DCFH-DA), 6-hydroxy-2,5,7,8-tetramethylchroman-2-carboxylic acid (Trolox), caffeic acid, ± 98% catechin, gallic acid, phosphate buffer saline (PBS), sodium chloride (NaCl), 3,3′,5,5′-tetramethylbenzidine (TMB), hydrogen peroxide (H_2_O_2_), Amberlite^®^ XAD 16, sodium hydroxide (NaOH), sodium dodecyl sulfate (SDS), 2,4-dinitrophenylhydrazine (DNPH), Cobalte (II) fluoride (CoF_2_), GSH and GSSG standards, orthophthalaldehyde (OPA), Cell Lytic™ M, protease inhibitor cocktail and rabbit anti-DNPH antibody were purchased from Sigma-Aldrich (St. Quentin Fallavier, France), Disodium fluorescein was obtained from TCI Europe (Antwerp, Belgium), Ferrous sulphate (FeSO_4_) was from Merck (Darmstadt, Germany), Folin reagent and hydrochloric acid (HCl) were from Panreac (Barcelona, Spain). CellTiter 96^®^ AQueous One Solution Cell Proliferation Assay was obtained from Promega (San Luis Obispo, CA, USA), and donkey anti-rabbit IgG HRP-labeled secondary antibody were obtained from Rockland (Gilbertsville, PA, USA). All cell culture media and supplements were obtained from Invitrogen (Gibco, Invitrogen Corporation, Paisley, UK).

Phenolic standards used were: neochlorogenic and chlorogenic acid, catechin, cyanidin-3-glucoside, cyanidin-3-rutinoside, peonidin-3-glucoside, procyanidin B2, quercetin-3-rutinoside, quercetin 3-*O*-glucoside and kaempferol-3-glucoside all from Extrasynthèse (Genay, France). Ultra-pure water (18.2 MΩ·cm) was obtained from a Millipore-Direct Q3 UV system (Millipore, Billerica, MA, USA).

### 3.2. Raw Material

Cherries of Saco cultivar were collected at Cova da Beira, Portugal, between May and June 2008 and stored at −18 °C. Raw material used in all extractions was obtained from whole fruit with seeds and stalks. Firstly, raw material was crushed in a domestic knife mill (UFESA, Lisbon, Portugal) followed by dehydration in a freeze drier (Freeze Dryer Modulyo, Edwards, UK) at −40 °C, in the absence of light. After 72 h, the raw material was milled in a grinder (Braun, KSM 2, Kronberg, Germany) and stored at −18 °C until the day of the experiment. 

### 3.3. Preparation of Cherry (Poly)phenolic-Rich Extract (Ch-PRE)

Powdered cherries were extracted with EtOH:H_2_O (1:1 *v*/*v*) solution (1:20, *w*/*v*), for 2 h, under continuous agitation (200 rpm, IKA dual-speed mixer), absent from light and at room temperature. The extract obtained was then filtered, centrifuged at 14,334 *g* for 10 min and the supernatant collected (solvent cherry extract).

Ch-PRE was prepared from the solvent cherry extract, performing a static adsorption process using a macroporous resin as previously reported by Serra *et al*., 2013 [23]. Taking into account the molecular weight and polarity of targeted compounds, the food grade macroporous resin, Amberlite^®^ XAD-16 was selected as adsorbent.

### 3.4. Phytochemical and Antioxidant Characterization

#### 3.4.1. Total Phenolic Content

Total concentration of phenolic compounds present in Ch-PRE was determined according to the Folin-Ciocalteau colorimetric method [54], as previously adapted by Serra *et al.* [13]. Results were expressed as means of triplicates ± SD (mg of gallic acid equivalents per g of dry extract—mg GAE·g^−1^ dry extract).

#### 3.4.2. HPLC-DAD-ED and LC-MS/MS Analysis

HPLC-DAD-ED: The High Performance Liquid Chromatography (HPLC) system used was a Thermo Finnigan (Surveyor model) equipped with an autosampler, pump, diode-array detector (DAD) and electrochemical detector (ED) (Dionex, ED40). Chromatographic separation was carried out on a Lichrocart RP-18 column (250 × 4 mm, 5 µm, Merck) in a thermostated oven at 35 °C. The injection volume was 20 µL. Diode array detector was programmed for a scanning between 200 and 800 nm at a speed of 1Hz with a bandwidth of 5 nm. The detection was monitored using three individual channels, 280, 360 and 527 nm, at a speed of 10 Hz with a bandwidth of 11 nm. Electrochemical detector was programmed to measure the signal by integrated voltammetry in potential ranges of −1.0 to 1.0V. The mobile phase used consisted of a gradient mixture of eluent A water:formic acid (99.9:0.1 *v*/*v*) and eluent B acetonitrile:water:formic acid (40:60:0.1 *v*/*v*/*v*). The following gradient of eluents was used: 0–15 min from 0% until 20% of eluent B; 10 min with 20% eluent B; 25–70 min, from 20% until 70% eluent B; 70–75 min, with 70% of eluent B; 75–85 min from 70% until 100% eluent B; 85–90 min, with 100% eluent B; 90–95 min from 100% to 0% of eluent B; and 95–100 min 100% of eluent A. The solvent flow rate was 0.7 mL·min^−1^. Chromoquest was used to control and acquire data.

All the compounds were quantified using standard solutions at different concentrations and results were expressed as mg·g^−1^ dw. Quantification of compounds was obtained using calibration curves of the available standards. At least seven concentration curves, ranging from 0.5 to 15 ppm were constructed from analytical standards, and each concentration point was injected in triplicate. Standard curves were all linear within the concentration range and linearity was ensured as *R*^2^ 0.97–1.000. It was estimated an error around 5%–6% in compounds quantification.

LC-MS/MS: An Alliance HPLC system (Waters 2695 Separation Module, Waters^®^, Milford, MA, USA) comprising a HPLC pump, photodiode array detector (DAD, Waters 2996) scanning from 210 to 600 nm and an autosampler cooled at 10 °C (Waters). Separations were carried out using a 100 × 2.0 mm i.d., 2.5 µm RP-18 column (Synergy, Max-RP from Phenomenex, Torrance, CA, USA), maintained at 35 °C. For chromatographic separation formic acid p.a. in Milli-Q^®^ water (0.5%) (Eluent A) and acetonitrile MS gradient grade (Eluent B) were used as the mobile phases at a flow rate of 0.3 mL·min^−1^. The elution program, was from 99% eluent A until 98% during the first 10 min; 5 min of isocratic elution with 98% of eluent A, a linear gradient during 72 min from 98% until 70% of eluent A. The injection volume was 10 µL. A mass spectrometer (MS/MS) Micromass Quattro Micro API, Waters^®^) with a Triple Quadrupole (TQ) and an electrospray ion source (ESI) operating in negative mode was used (Waters). Analyses were carried out using full scan from *m*/*z* 100 to 1000. Source temperature was 120 °C and desolvation temperature was 350 °C. Cone gas flow and desolvation gas flow were 50 and 750 L/Hr respectively. Different collision energy values were used in order to obtain fragmentation spectra of detect fragment ions. Argon was used as collision gas. The capillary voltage was 3.0 kV and cone voltage 30 V. MassLynx software (version 4.1, Waters^®^) was used to control and acquire data.

### 3.5. Oxygen Radical Absorbance Capacity (ORAC)

Peroxyl radical scavenging capacity was determined by the ORAC method. The assay was carried out by following method of Huang *et al.* [55] modified for the FL800 microplate reader (BioTek Instruments, Winooski, VT, USA), as described by Feliciano *et al.* [29]. This assay measured the ability of the antioxidant species in the sample to inhibit the oxidation of disodium fluorescein (FL) catalysed by peroxyl radicals generated from AAPH. All data were expressed as means of micromoles of trolox equivalent antioxidant capacity per gram of dry extract (µmol TEAC·g^−1^ dw) ± SD.

### 3.6. Hydroxyl Radical Adverting Capacity (HORAC)

HORAC assay was based on a previously reported method [56] modified for the FL800 microplate fluorescence reader, as described by Serra *et al.* [19]. This assay evaluates the hydroxyl radical prevention capacity of a sample using fluorescein (FL) as probe. Caffeic acid was used as standard and data were expressed as means of micromoles of caffeic acid equivalents per gram of dry extract (µmol CAE·g^−1^ dw) ± SD.

### 3.7. Cell-Based Assays

#### 3.7.1. Cell Culture

Human colon carcinoma Caco-2 cells were purchased from Deutsche Sammlung von Mikroorganismen und Zellkulturen (DSMZ, Braunschweig, Germany), and were routinely grown in RPMI 1640 supplemented with 10% (*v*/*v*) of heat-inactivated foetal bovine serum (FBS), 2 mM of glutamine and 1% of 5000 U of penicillin-streptomycin solution. Stock cells were maintained as monolayers in 175 cm^2^ culture flasks. Cells were subcultured every week at a split ratio of 1:4 by treatment with 0.1% trypsin and 0.02% EDTA and incubated at 37 °C in a 5% CO_2_ humidified atmosphere.

Human SK-N-MC neuronal cells were obtained from the European Collection of Cell Cultures (ECACC) and cultured in DMEM supplemented with 2 mM glutamine, 10% (*v*/*v*) FBS, 1% (*v*/*v*) non-essential amino acids, and 1 mM sodium pyruvate containing 50 U·mL^−1^ of penicillin and 50 μg·mL^−1^ of streptomycin. The cells were maintained at 37 °C in 5% CO_2_ and split at sub-confluence of 70%–80%, using 0.05% trypsin/EDTA.

Ch-PRE toxicity in cells were performed for both cell lines: Caco-2 and SK-N-MC cells using CellTiter^®^ Reagent accordingly to manufacturer instructions for a concentrations range of 0–250 μg GAE of Ch-PRE per mL of cell medium as previously described [23,43].

#### 3.7.2. Intracellular Reactive Oxygen Species (ROS)

To evaluate the intracellular antioxidant capacity of Ch-PRE, Caco-2 and SK-N-MC cells were used and the formation of intracellular ROS was monitored using the fluorescent probe, DCFH-DA according each cell model particularities.

Intestinal Epithelial cell model (Caco-2 cells): cells were seeded at a density of 2 × 10^4^ cells/well in 96-well plates and the medium was changed every 48 h. On this model, cellular antioxidant activity of Ch-PRE was evaluated pre- and co-incubating the extract with a stress inducer (H_2_O_2_) and following the ROS formation in confluent monolayers of Caco-2 cells. Briefly, in the case of pre-incubation, Caco-2 cells were washed with PBS and incubated with Ch-PRE (50 µg GAE·mL^−1^) and 100 μM of DCFH-DA for 1 h. Then, the medium was removed and cells were washed with PBS followed by the addition of 10 mM of H_2_O_2_ for 1 h. For the co-incubation assay, cells were incubated with 100 μM of DCFH-DA for 1 h. Then the medium with DCFH-DA was removed and cells were washed with PBS followed by the addition of Ch-PRE (50 µg GAE·mL^−1^) simultaneously with 10 mM of H_2_O_2_ for 1 h.

Neurodegeneration cell model: SK-N-MC cells were seeded in 96-well plates at a density of 1.25 × 10^4^ cells per well. After 24 h, SK-N-MC cells were pre-incubated with various concentrations of Ch-PRE for 2 or 24 h, to cover different timescale events. After pre-incubation, cells were washed with PBS and incubated with 25 μM DCFH-DA in PBS for 30 min at 37 °C. Cells were washed and H_2_O_2_ (300 μM) in PBS, was added for 1 h.

Fluorescence was measured (λ_ex_: 485 nm, λ_em_: 530 nm) using a FLx800 Fluorescence Microplate Reader (Biotek) for 1 h at 37 °C. ROS generation was calculated as an increase in fluorescent signal between control and H_2_O_2_-treated cells.

#### 3.7.3. Determination of Protein Carbonyl Content on Intestinal Epithelial Cells

Caco-2 cells were seeded in 6-well plate at a density of 1.0 × 10^5^ cells/well and cultured in standard medium (RPMI-1640 supplemented with 2 mM glutamine, 10% (*v*/*v*) FBS and 1% penicillin-streptomycin) during 21 days to obtain fully differentiated cells. Cells were washed with PBS and pre-incubated with Ch-PRE (50 µg GAE·mL^−1^) diluted in PBS for 1 h (except in control wells that were incubated only with PBS). Then, Ch-PRE were removed and 10 mM of H_2_O_2_ (stress inductor) was added, except to control well (without Ch-PRE and without stress inducer) for 1 h. In co-incubation assay, 50 µg GAE·mL^−1^ of Ch-PRE and 10 mM of H_2_O_2_ were added simultaneously for 1 h. The media was removed and 200 µL of Cell Lytic™ supplemented with a proteases inhibitor cocktail was added for 5 min. Cells were removed by scrapping and centrifuged at 14,000 *g*, for 10 min at 4 °C (Hettich Zentrifugen MIKRO 220R, Hettich, Tuttlingen, Germany) and supernatants were freeze at −80 °C until proteins carbonyl determination.

Carbonylated proteins were determinate according to Ramful *et al.* [57] with slight modifications previously reported [24]. Experiments were done in triplicate and results are expressed as a percentage of the absorbance compared to control cells.

#### 3.7.4. Glutathione (GSH) and Glutathione Disulfide (GSSG) Quantification on Intestinal Epithelial Cells

For GSH and GSSG quantification, Caco-2 cells were seeded in 6-well plates as mentioned above on Section 3.7.3. The media was removed and cells were detached by adding trypsin followed by inactivation with cell medium and centrifugation at 14,000 *g*, for 10 min at 4 °C (Hettich Zentrifugen MIKRO 220R). Cells were resuspended in PBS and centrifuged at 14,000 *g*, for 10 min at 4 °C (Hettich Zentrifugen MIKRO 220R).

GSH and GSSG quantification assay was based on a previously reported method [58] as adapted by Tavares *et al.* [46].

#### 3.7.5. Evaluation of Neuroprotective Effect

To evaluate the neuroprotective activity of Ch-PRE, a neurodegeneration cell model was used as described by Tavares *et al.* [46]. SK-N-MC cells were treated with H_2_O_2_ to induce approximately 50% of cell death [46]. Briefly, cells were seeded at 2.5 × 10^4^ cells per well. After 24 h of growth, the medium was removed and the cells were washed with PBS. Cells were pre-incubated with non-toxic concentrations of Ch-PRE extract for 24 h. Cells were washed again with PBS and medium was replaced by medium containing 0.5% (*v*/*v*) FBS and 300 μM H_2_O_2_ for 24 h. Cell viability was monitored with CellTiter-Blue^®^ Reagent accordingly to instructions and as previously described by Tavares *et al.* [46].

### 3.8. Statistical Analysis

All data are expressed as means ± standard deviation (SD) and individual experiments were performed at least in triplicate. The statistical analysis was completed using SigmaStat 3.0^®^ software (3.0 version, Systat Software Inc., San Jose, CA, USA). All values were tested for normal distribution and equal variance. When homogeneous variances were confirmed, data were analyzed by One Way Analysis of Variance (ANOVA) coupled with the *post-hoc* Tukey test to identify means with significant differences. Paired comparisons were done by *t*-tests.

## 4. Conclusions

In this study, the antioxidant properties of a cherry extract (Ch-PRE) derived from fruit surpluses (wastes) was investigated using two different human cell lines: intestinal epithelial cells (Caco-2 cells) and a neuronal cells (SK-N-MC cells). Results showed that by using a combination of a green solid–liquid extraction and a static adsorption process with food grade macroporous resins it was possible to obtain a poly(phenolic)-rich extract with high ORAC and HORAC value. The main antioxidant compounds identified included neochlorogenic acid, quercetin-3-rutinoside and anthocyanins, namely cyanidin-3-glucoside. At a cellular level, Ch-PRE demonstrated to be effective in reducing intracellular ROS production in both human cell lines. Additionally, in the intestinal cell model, cherry extract seems to be involved in modulation of endogenous antioxidant system and reduction/prevention of protein oxidation. In the neuronal cell model, cherry extract is effective in preventing cell death induced by oxidative stress. Altogether, the results obtained suggest that Ch-PRE is an attractive candidate to be applied and formulated as a high added value ingredient, such as a nutraceutical agent, for the prevention of oxidative stress-induced disorders such as intestinal inflammation disorders or, with an appropriate delivery system, for neurodegenerative diseases. However, further studies are required in order to deeply understand the mechanisms, doses and role of each Ch-PRE compound on specific disorders biomarkers.

## Figures and Tables

**Table 1:** Phenolic compounds from Ch-PRE. Peak assignment accordingly profile in Figure 1, retention time (RT), mass spectral and concentration. Compounds identified by LC-MS/MS.

Peak	RT (min)	[M − H]^−^ *m*/*z* ^1^	MS/MS *m*/*z* ^1^	Assigned Identity	Concentration ^2^ (mg·g^−1^ dw)
1	18.28	353	MS^2^ [353]: 135, 179, 195	Neochlorogenic acid	19.49
2	23.43	289	MS^2^ [289]: 245, 205, 125, 109	Catechin	6.66
3	26.33	353	MS^2^ [353]: 135, 179, 195	Chlorogenic acid	1.83
4	33.97	577	MS^2^ [577]: 289, 125	Procyanidin B2	0.98
8	51.91	609	MS^2^ [609]: 301	Quercetin-3-rutinoside	17.00
9	52.85	463	MS^2^ [463]: 301	Quercetin-3-glucoside	1.74
10	58.01	447	MS^2^ [447]: 285	Kaempferol-3-glucoside	0.97
11	72.34	447	MS^2^ [447]: 285	Sakuranin	*
12	92.12	285	MS^2^ [285]: 243, 164	Isosakuranetin	*

^1^ Detection of [M − H]^−^ and fragmentation experiments for identification were performed in the LC-MS/MS system; ^2^ Quantification experiments were performed in the HPLC-DAD-ED equipment, and conditions of analysis were as described in the Experimental Section; * Tentatively identified without using standards.

**Table 2:** Anthocyanins identified on Ch-PRE. Peak assignment accordingly profile in Figure 1A, retention time (RT). Compound identified and quantified by HPLC-DAD-ED using standards.

Peak	RT (min)	Assigned Identity	Concentration (mg·g^−1^ dw)
5	44.73	Cyanidin-3-glucoside	14.04
6	46.56	Cyanidin-3-rutinoside	83.50
7	51.11	Peonidin-3-glucoside	1.60

## References

[1] Halliwell B. (2011). Free radicals and antioxidants—Quo vadis?. Trends Pharmacol. Sci..

[2] Obrenovich M.E., Li Y., Parvathaneni K., Yendluri B.B., Palacios H.H., Leszek J., Aliev G. (2011). Antioxidants in health, disease and aging. CNS Neurol. Disord. Drug Targets.

[3] Rodrigo R., Miranda A., Vergara L. (2011). Modulation of endogenous antioxidant system by wine (poly)phenols in human disease. Clin. Chim. Acta.

[4] Willcox J., Ash S., Catignani G. (2004). Antioxidants and prevention of chronic disease. Crit. Rev. Food Sci. Nutr..

[5] Kaplan M., Mutlu E.A., Benson M., Fields J.Z., Banan A., Keshavarzian A. (2007). Use of herbal preparations in the treatment of oxidant-mediated inflammatory disorders. Complement Ther. Med..

[6] Rahman I., Biswas S.K., Kirkham P.A. (2006). Regulation of inflammation and redox signaling by dietary(poly)phenols. Biochem. Pharmacol..

[7] Rahimi R., Mozaffari S., Abdollahi M. (2009). On the use of herbal medicines in management of inflammatory bowel diseases: A systematic review of animal and human studies. Dig. Dis. Sci..

[8] Ebrahime A., Scluesener H. (2012). Natural (poly)phenols against neurodegenerative disorders: Potentials and pitfalls. Ageing Res. Rev..

[9] Macedo D., Tavares L.R., McDougall G.J., Miranda H.V., Stewart D., Ferreira R.B., Tenreiro S., Outeiro T.F., Santos C.N. (2015). (Poly)phenols protect from α-synuclein toxicity by reducing oxidative stress and promoting autophagy. Hum. Mol. Genet..

[10] McCune L., Kubota C., Stendell-Hollis N., Thomson C. (2011). Cherries and Health: A Review. Crit. Rev. Food Sci. Nutr..

[11] Ship J., Abdel-Aal E.M. (2010). Food Applications and Physiological Effects of Anthocyanins as Functional Food Ingredients. Open Food Sci. J..

[12] Miguel M.G. (2011). Anthocyanins: Antioxidant and/or anti-inflammatory activities. J. Appl. Pharm. Sci..

[13] Serra A., Duarte R., Bronze M., Duarte C. (2011). Identification of bioactive response in traditional cherries from Portugal. Food Chem..

[14] Ferretti G., Bacchetti T., Belleggia A., Neri D. (2010). Cherry antioxidants: From farm to table. Molecules.

[15] Kappel F., FisherFleming B., Hogue E. (1996). Fruit characteristics and sensory attributes of an ideal sweet cherry. HortScience.

[16] Predieri A., Dris R., Rapparini F. (2004). Influence of growing conditions on yield and quality of cherry: II. Fruit quality. J. Food Agric. Environ..

[17] Goncalves B., Landbo A.K., Knudsen D., Silva A.P., Moutinho-Pereira J., Rosa E., Meyer A.S. (2004). Effect of ripeness and postharvest storage on the phenolic profiles of cherries (*Prunus avium* L.). J. Agric. Food Chem..

[18] Serra A.T., Seabra I.J., Braga M.E.M., Bronze M.R., de Sousa H.C., Duarte C.M.M. (2010). Processing cherries (*Prunus avium*) using supercritical fluid technology. Part 1: Recovery of extract fractions rich in bioactive compounds. J. Supercrit. Fluids.

[19] Serra A.T., Matias A.A., Almeida A.P.C., Bronze M.R., Alves P.M., de Sousa H.C., Duarte C.M.M. (2011). Processing cherries (*Prunus avium*) using supercritical fluid technology. Part 2. Evaluation of SCF extracts as promising natural chemotherapeutical agents. J. Supercrit. Fluids.

[20] Kammerer D.R., Saleh Z.S., Carle R., Stanley R.A. (2007). Adsorptive recovery of phenolic compounds from apple juice. Eur. Food Res. Technol..

[21] Kammerer J., Carle R., Kammerer D.R. (2011). Adsorption and Ion Exchange: Basic Principles and Their Application in Food Processing. J. Agric. Food Chem..

[22] Soto M.L., Moure A., Domínguez H., Parajó J.C. (2011). Recovery, concentration and purification of phenolic compounds by adsorption: A review. J. Food Eng..

[23] Serra A.T., Poejo J., Matias A.A., Bronze M.R., Duarte C.M.M. (2013). Evaluation of Opuntia spp. derived products as antiproliferative agents in human colon cancer cell line (HT29). Int. Food Res..

[24] Matias A.A., Nunes S., Poejo J., Mecha E., Serra A.T., Bronze M.R., Duarte C.M.M. (2014). Antioxidant and anti-inflammatory activity of a flavonoid-rich concentrate recovered from Opuntia ficus-indica juice. Food Funct..

[25] Schaefer S., Baum M., Eisenbrand G., Janzowski C. (2006). Modulation of oxidative cell damage by reconstituted mixtures of phenolic apple juice extracts in human colon cell lines. Mol. Nutr. Food Res..

[26] Serra A.T. (2010). Valorization of Traditional Portuguese Apples and Cherries—Biochemical Characterization and Development of Functional Ingredients. Ph.D. Thesis.

[27] Piccolella S., Fiorentino A., Pacifico S., D’Abrosca B., Uzzo P., Monaco P. (2008). Antioxidant properties of sour cherries (*Prunus cerasus* L.): Role of colorless phytochemicals from the methanolic extract of ripe fruits. J. Agric. Food Chem..

[28] Tavares L., Carrilho D., Tyagi M., Barata D., Serra A.T., Duarte C.M.M., Duarte R.O., Feliciano R.P., Bronze M.R., Chicau P. (2010). Antioxidant Capacity of Macaronesian Traditional Medicinal Plants. Molecules.

[29] Feliciano R.P., Bravo M.N., Pires M.M., Serra A.T., Duarte C.M., Boas L.V., Bronze M.R. (2009). Phenolic Content and Antioxidant Activity of Moscatel Dessert Wines from the Setubal Region in Portugal. Food Anal. Method.

[30] Langerholc T., Maragkoudakisb P.A., Wollgastb J., Gradisnikc L., Cencica A. (2011). Novel and established intestinal cell line models—An indispensable tool in food science and nutrition. Trends Food Sci. Technol..

[31] Williamson G., Manach C. (2005). Bioavailability and bioefficacy of (poly)phenols in humans. II. Review of 93 intervention studies. Am. J. Clin. Nutr..

[32] Yi W., Akoh C.C., Fischer J., Krewer G. (2006). Absorption of anthocyanins from blueberry extracts by Caco-2 human intestinal cell monolayers. J. Agric. Food Chem..

[33] Deprez S., Mila I., Huneau J.F., Tome D., Scalbert A. (2001). Transport of proanthocyanidin dimer, trimer, and polymer across monolayers of human intestinal epithelial Caco-2 cells. Antioxid. Redox Signal..

[34] Farrell T.L., Dew T.P., Poquet L., Hanson P., Williamson G. (2011). Absorption and metabolism of chlorogenic acids in cultured gastric epithelial monolayers. Drug Metab. Dispos..

[35] Konishi Y., Kobayashi S. (2004). Transepithelial transport of chlorogenic acid, caffeic acid, and their colonic metabolites in intestinal Caco2 cell monolayers. J. Agric. Food Chem..

[36] Fraga C.G., Galleano M., Verstraeten S.V., Oteiza P.I. (2010). Basic biochemical mechanisms behind the health benefits of (poly)phenols. Mol. Aspects Med..

[37] Dalle-Donne I., Rossi R., Giustarini D., Milzani A., Colombo R. (2003). Protein carbonyl groups as biomarkers of oxidative stress. Clin. Chim. Acta.

[38] Ballatori N., Krance S.M., Notenboom S., Shi S., Tieu K., Hammond C.L. (2009). Glutathione dysregulation and the etiology and progression of human diseases. Biol. Chem..

[39] Kasthuri B., Magalingam A., Kutty R., Nagaraja H. (2015). Protective Mechanisms of Flavonoids in Parkinson’s Disease. Oxid. Med. Cell. Longev..

[40] Viljanen K., Kylli P., Hubbermann E.M., Schwarz K., Heinonen M. (2005). Anthocyanins antioxidant activity and partition behavior in Whey protein emulsion. J. Agric. Food Chem..

[41] Schmitt-Schillig S., Schaffer S., Weber C.C., Eckert G.P., Müller W.E. (2005). Flavonoids and the aging brain. J. Physiol. Pharmacol..

[42] Youdim K.A., Shukitt-Hale B., Joseph J.A. (2004). Flavonoids and the brain: Interactions at the blood-brain barrier and their physiological effects on the central nervous system. Free Radic. Biol. Med..

[43] Tavares L., Figueira I., McDougall G.J., Vieira H.L., Stewart D., Alves P.M., Ferreira R.B., Santos C.N. (2013). Neuroprotective effects of digested (poly)phenols from wild blackberry species. Eur. J. Nutr..

[44] Cavazzoni M., Barogi S., Baracca A., Parenti Castelli G., Lenaz G. (1999). The effect of aging and an oxidative stress on peroxide levels and the mitochondrial membrane potential in isolated rat hepatocytes. FEBS Lett..

[45] Tabner B.J., El-Agnaf O.M., Turnbull S., German M.J., Paleologou K.E., Hayashi Y., Cooper L.J., Fullwood N.J., Allsop D. (2005). Hydrogen peroxide is generated during the very early stages of aggregation of the amyloid peptides implicated in Alzheimer disease and familial British dementia. J. Biol. Chem..

[46] Tavares L., Figueira I., Macedo D., McDougall G.J., Leitão M.C., Vieira H.L.A., Stewart D., Alves P.M., Ferreira R.B., Santos C.N. (2012). Neuroprotective effect of blackberry (*Rubus* sp.) (poly)phenols is potentiated after simulated gastrointestinal digestion. Food Chem..

[47] Manach C., Williamson G., Morand C., Scalbert A., Remesy C. (2005). Bioavailability and bioefficacy of (poly)phenols in humans. I. Review of 97 bioavailability studies. Am. J. Clin. Nutr..

[48] .Lu Y.H., Su M.Y., Huang H.Y., Lin L., Yuan C.G. (2010). Protective effects of the citrus flavanones to PC12 cells against citotoxicity induced by hydrogen peroxide. Neurosci. Lett..

[49] Pavlica S., Gebhardt R. (2010). Protective effects of flavonoids and two metabolites against oxidative stress in neuronal PC12 cells. Life Sci..

[50] Tarozzi A., Morroni F., Hrelia S., Angeloni C., Marchesi A., Cantelli-Forti G., Hrelia P. (2007). Neuroprotective effects of anthocyanins and their *in vivo* metabolites in SH-SY5Y cells. Neurosci. Lett..

[51] Chen G., Luo J. (2010). Anthocyanins: Are they beneficial in treating ethanol neurotoxicity?. Neurotox. Res..

[52] Min J., Yu S.W., Baek S.H., Nair K.M., Bae O.N., Bhatt A., Kassab M., Nair M.G., Maiid A. (2011). Neuroprotective effect of cyanidin-3-*O*-glucoside anthocyanin in mice with focal cerebral ischemia. Neurosci. Lett..

[53] Son T.G., Camandola S., Mattson M.P. (2008). Hormetic dietary phytochemicals. Neuromol. Med..

[54] Singleton V.L., Rossi J.A. (1965). Colorimetry of total phenolics with phosphomolybdic-phosphotungstic acid reagents. Am. J. Enol. Vitic..

[55] Huang D., Ou B., Hampsch-Woodill M., Flanagan J., Prior R. (2002). High-throughput assay of oxygen radical absorbance capacity (ORAC) using a multichannel liquid handling system coupled with a microplate flourescence reader in 96-well format. J. Agric. Food Chem..

[56] Ou B., Hampsch-Woodill M., Flanagan J., Deemer E., Prior R., Huang D. (2002). Novel fluorometric assay for hydroxyl radical prevention capacity using fluorescein as the probe. J. Agric. Food Chem..

[57] Ramful D., Tarnus E., Rondeau P., da Silva C.R., Bahorun T., Bourdon E. (2010). Citrus Fruit Extracts Reduce Advanced Glycation End Products (AGEs)- and H_2_O_2_-Induced Oxidative Stress in Human Adipocytes. J. Agric. Food Chem..

[58] Kand’ar R., Zakova P., Lotkova H., Kucera O., Cervinkova Z. (2007). Determination of reduced and oxidized glutathione in biological samples using liquid chromatography with fluorimetric detection. J. Pharm. Biomed. Anal..

